# Development of a simple, rapid, and sensitive molecular diagnostic assay for cholera

**DOI:** 10.1371/journal.pntd.0011113

**Published:** 2023-02-06

**Authors:** Subhra Chakraborty, Mirza Velagic, Sean Connor

**Affiliations:** Department of International Health, Johns Hopkins Bloomberg School of Public Health, Baltimore, Maryland, United States of America; Wayne State University, UNITED STATES

## Abstract

Cholera continues to inflict high rates of morbidity and mortality. Prompt identification of cholera cases facilitates rapid outbreak responses in the short term while providing reliable surveillance data to guide long-term policies and interventions. Microbiological stool culture, the current recognized gold standard for diagnosing cholera, has significant limitations. Rapid diagnostic tests (RDTs) represent promising alternatives for diagnosing cholera in areas with limited laboratory infrastructure. However, studies conducted with the current cholera RDTs demonstrated wide variations in sensitivity and specificity. To address this gap in the diagnosis of cholera, we developed a simple, rapid, and sensitive diagnostic assay, "Rapid LAMP based Diagnostic Test (RLDT)." With a novel, simple sample preparation method directly from the fecal samples along with lyophilized reaction strips and using established Loop-mediated Isothermal Amplification (LAMP) platform, cholera toxin gene (*ctxA*) and O1 (O1*rfb*) gene could be detected in less than an hour. Cholera RLDT assay is cold chain and electricity-free. To avoid any end-user bias, a battery-operated, handheld reader was used to read the RLDT results. The performance specifications of the cholera RLDT assay, including analytical sensitivity and specificity, were evaluated using direct fecal samples, dried fecal samples on filter paper, and environmental water samples spiked with cholera strain. The limit of detection (LOD) was ~10^4^ CFU/gm of stool for both *ctxA* and O1 genes, corresponding to about 1 CFU of *Vibrio cholerae* per reaction within 40 minutes. The LOD was 10 bacteria per ml of environmental water when tested with RLDT directly, without enrichment. Being simple, RLDT has the potential to be applied in resource-poor endemic settings for rapid, sensitive, and reliable diagnosis of cholera.

## Author summary

Cholera is an acute diarrheal infection caused by *Vibrio cholerae*. Cholera continues to severely threaten the health of the impoverished and vulnerable populations in the endemic regions. The present laboratory-based cholera detection methods take time and require well-equipped facilities and well-trained staff that are often not available in the resource-poor settings. The available rapid diagnostic tests have a wide range of sensitivity and specificity. A rapid, simple, sensitive, and reliable diagnostic for cholera is required, which would identify cholera cases early on in outbreaks to facilitate a rapid response and provide reliable surveillance data. We developed a simple, rapid, and sensitive diagnostic assay, "Rapid LAMP based Diagnostic Test (RLDT)," to detect cholera in low-resource settings. Using RLDT, cholera could be detected directly from stool, dried stool on filter paper, and environmental water in less than one hour with minimum hands-on time. The RLDT assay does not require maintaining a cold chain and is electricity-free, facilitating point-of-care testing. The RLDT assay is sensitive, simple to use, and can be scaled up for rapid detection of cholera and surveillance in low-resource endemic settings.

## Introduction

Cholera is an acute, secretory diarrheal disease caused by *Vibrio cholerae* [1,2]. There are an estimated 1.3 to 4.0 million cholera cases annually, leading to 21,000–143,000 deaths [3,4]. Approximately 1.3 billion people living in 69 countries are at risk of contracting the disease [5]. Cholera epidemics have been increasing in frequency, intensity, and duration, indicating more effective approaches to prevention and control are required [6]. Rapid, sensitive, and reliable diagnostic assays are needed to rapidly contain cholera outbreaks and get surveillance data to guide long-term policies and interventions.

The current gold standard for detection of *V*. *cholerae* is culturing stool or rectal swabs on the selective media [thiosulphate citrate bile salts sucrose (TCBS), or taurocholate-tellurite gelatin agar (TTGA)] either directly or after enrichment in alkaline peptone water (APW), followed by serotyping and bio-typing with antisera [7–9]. The culture method requires competent laboratory support and technical skills, which are not always available at health facilities or laboratories where cholera is endemic. In addition, this method is not sensitive and can take up to 3 days after collection of fecal specimens, which is not ideal for outbreak situations.

Molecular methods like PCR or quantitative PCR are not widely used for cholera diagnostics or surveillance. Several methods are developed by the laboratories, but a standard, validated technique is not yet available. PCR requires trained technicians, a well-equipped laboratory, and a supply of reagents that are not available outside the central laboratories.

Several cholera Rapid Diagnostic Tests (RDTs) have been developed. The commonly used RDTs are based on the lateral flow that detects the lipopolysaccharide of *V*. *cholerae* O1 and O139 by immunochromatographic assay [10]. Although these tests are well suited to meet the demand for cholera diagnosis in areas where culture or PCR-based methods are not feasible, there are also limitations. A systematic review of diagnostic tests for cholera demonstrated a notable absence of evidence to support the use of these rapid tests [11]. Several studies conducted with Crystal VC, the most widely used RDT, demonstrated suboptimal performance characteristics [9,11]; for example, Crystal VC has demonstrated a wide variation of sensitivity (ranging from 58%–100%) and specificity (ranging from 60%–100%) [11–21]. The performance of Crystal VC improved if tested after enrichment in 1% APW for six hours [22,23]. A study in Haiti [24] which compared Crystal VC, Artron, and SD Bioline RDTs, reported that the performance characteristics of all three RDTs evaluated in this study did not meet the expected minimal performance of a sensitivity of at least 90% and a specificity of at least 85% recommended by the Global Task Force for cholera Control [21].

Cholera is transmitted through water contaminated with *Vibrio cholerae*. We previously adapted the Crystal VC dipstick assay and developed a simple technique for identifying cholera in water samples [25]. However, this assay requires incubation in alkaline peptone water for 24 hours. A rapid and sensitive assay would be valuable to quickly identify if any water sources are contaminated with cholera, and therefore response measures can be taken to contain the transmission.

To address these concerns, we developed a simple and sensitive rapid detection test, "Rapid LAMP-based Diagnostic Test (RLDT)," mostly equipment, cold chain, and electricity-free. We tested the performance specifications including sensitivity, specificity, stability, repeatability, and reproducibility of the cholera RLDT assay using fresh stool, dried stool on filter paper and from environmental water. We believe the RLDT assay has the potential to be applied for cholera case detection and surveillance in patients and water in cholera endemic and epidemic regions of the world.

## Methods

### Optimization of cholera RLDT

The RLDT assay includes a novel sample preparation method and is based on the Loop mediated Isothermal Amplification (LAMP) technology, which is modified to apply to resource-poor settings. The LAMP is an isothermal nucleic acid amplification method first developed by Notomi *et al*. [26]. The LAMP technique is based on auto cycling and high DNA strand displacement activity through repeated elongation reactions occurring at the loop regions [26].

We developed RLDT to detect *V*. *cholerae* using OmniAmp 2X Isothermal Master Mix (Lucigen, WI). The final concentrations of the reaction mixtures were 1X OmniAmp Mix, 1X LAMP primer mix, 2 mM Fiona Green dye (Marker Gene, OR), and 5 μL of DNA, with enough molecular grade DNase-RNase-free water added to bring the total reaction volume to 25 μL. Primers were designed to target selected regions within the *rfb* gene cluster specific for the O1 lipopolysaccharide of the *V*. *cholerae* and cholera toxin subunit A (*ctxA*). Since the cholera toxin gene and the heat-labile toxin (LT) gene of enterotoxigenic *E*. *coli* (ETEC) are similar in their sequence, the primers were designed to detect *ctxA* specifically and not LT. Primer Explorer V5 software was employed to design potential primers from which a set of 3 primer pairs consisting of two outer primers (labeled as forward primer F3 and backward primer B3), two inner primers (labeled as forward inner primer FIP and backward inner primer BIP), and two loop primers (forward loop primer LF and backward loop primer LB) were selected for further testing. Following selection, primer specificity was assessed against sequences in the GenBank database using the National Center for Biotechnology Information’s Basic Local Alignment Search Tool (BLAST). The primer sequences, concentrations, and GenBank IDs are shown in Table 1. Optimization experiments were initially performed on a Step One Plus Real-Time PCR System (Applied Biosystems, CA), where amplification was recorded by detecting fluorescence and measured by the instrument software. Time to Result (TTR) calculations were performed by setting the threshold at 4000 reflective fluorescence units (RFU). The amplification reaction was performed at 71°C for 40 minutes. Later experiments and assays with RLDT kits were performed using a real-time isothermal fluorometer (reader) (AmpliFire, Agdia Inc., IN). To enhance stability and enable storage at ambient temperatures, a complete 1X OmniAmp-LAMP formulation and 10% trehalose (Sigma-Aldrich, MO) was dispensed into 0.2 ml microcentrifuge tubes in strips, and the mixture was lyophilized at the Johns Hopkins University core laboratory using a Labconco FreeZone bench-top lyophilizer (Labconoco Corporation, MO). The lyophilized reaction tube (LRT) strips were packaged into light-resistant bags containing a desiccant pouch. While performing the assay, prepared samples were added to LRT strips to rehydrate the reagents and bring the total volume in each tube to 25μl. The strips were placed in the reader to detect *O1 rfb* and *ctxA* target genes.

**Table 1:** List of cholera RLDT primers used in this study.

O1*rfb*	GenBank: X59554	
Primer Name	Sequence (5’ - 3’)	Primer Concentration
F3	TCTTCTGCTACCAGTGGCGTAC	0.2 μM
B3	TTCAAGTGGAGCACTTGGGCTA	0.2 μM
FIP	TGCAAAACGGGCGACGTTTAGGCTCGTCGACAGACTCGAGCA	1.6 μM
BIP	TGATCCGACAAGCCCAAATGCCACTCGATGTTGAGGCGAAGTTTAGGT	1.6 μM
LF	AACACCTCCTGCATAACTCTTGC	0.8 μM
LB	GCTCGTATTGCGGCGGTAA	0.8 μM
***ctxA***	GenBank: AF452584	
**Primer Name**	**Sequence (5’ - 3’)**	**Primer Concentration**
F3	GTTATATCGGGCAGATTCTAG	0.2 μM
B3	GTTTGACCCACTAAGTGG	0.2 μM
FIP	TTTGAGTACCTCGGTCAAAGTACACCTCCTGATGAAATAAAGC	1.6 μM
BIP	TGATCATGCAAGAGGAACTCAGATTGAGGTGGAAACATATCC	1.6 μM
LF	TCTGTCCTCTTGGCATAAGACCA	0.8 μM
LB	CGGGATTTGTTAGGCACGATGA	0.8 μM

### Sample preparation for cholera RLDT

#### Stool samples

**Fig 1:**
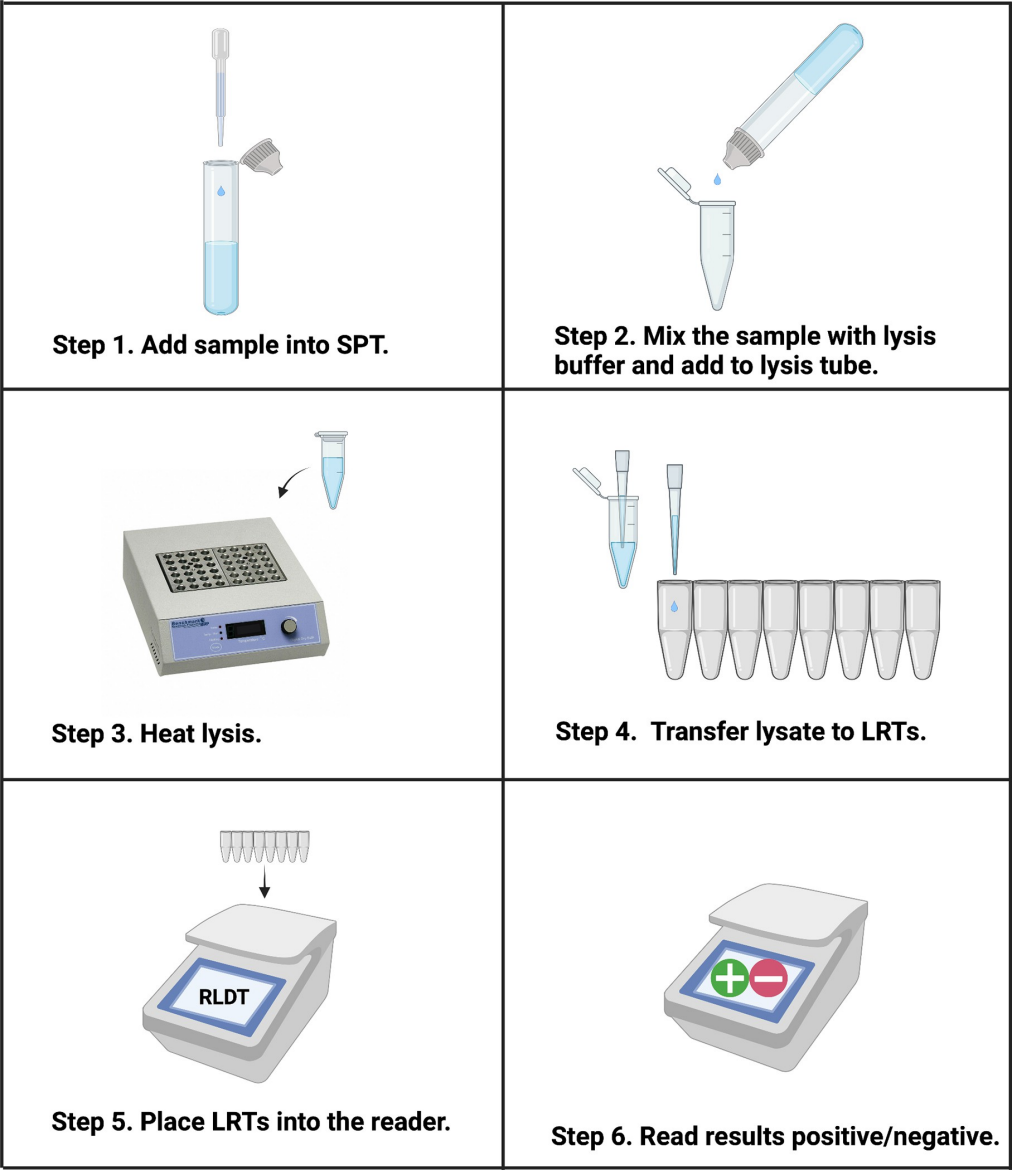
Workflow of cholera RLDT using RLDT kit. Step 1, using the fixed volume pipette provided in the kit, add a stool sample to the Sample Processing Tube (SPT) with lysis buffer. Step 2, Cap the SPT tube and mix by inverting the tube up and down. Squeeze the SPT tube to add the lysis buffer + stool sample to the lysis tube. Step 3, lysis tube is heated for 5 minutes in a heat block or water bath. Step 4, add 25 μL of lysate to each lyophilized reaction tube (LRT) strip tube using a disposable fixed volume pipette. Step 5, place the LRTs in the reader. Scan the barcode for the cholera RLDT program. Step 6, read the results displayed on the screen real time or after 40 minutes. All the reagents and sample processing accessories required for RLDT are included in the kit. The illustration was created with BioRender.com https:doi.org/10.1371/journal.pntd.0010180.g001.

We previously developed a simple, rapid, and sensitive method for sample preparation directly from stool to facilitate using the RLDT assay in low resource settings [27]. We adapted the method for the detection of cholera. Stool samples were diluted into an extraction lysis buffer using a sample preparation tube (SPT), and heat lysis was performed to release nucleic acids into the solution prior to amplification (Fig 1). An assay inhibitor control, consisting of the M13mp18 plasmid (New England BioLabs, MA), was included in the extraction buffer, and the lysates were used as templates in the RLDT reactions.

### Dried stool spots on filter paper

We also adapted our previously developed sample preparation method [27] to detect *V*. *cholerae* from the stool samples dried on filter papers. Fifty microliters of stool samples were placed on Whatman 903 Protein Saver Cards (Millipore, Sigma, MO, USA) and left to dry overnight. The following day, dried samples on the cards were cut out with sterile scissors, added to the SPT tube, and processed as described before directly from the stool (Fig 1).

### Water samples

**Fig 2:**
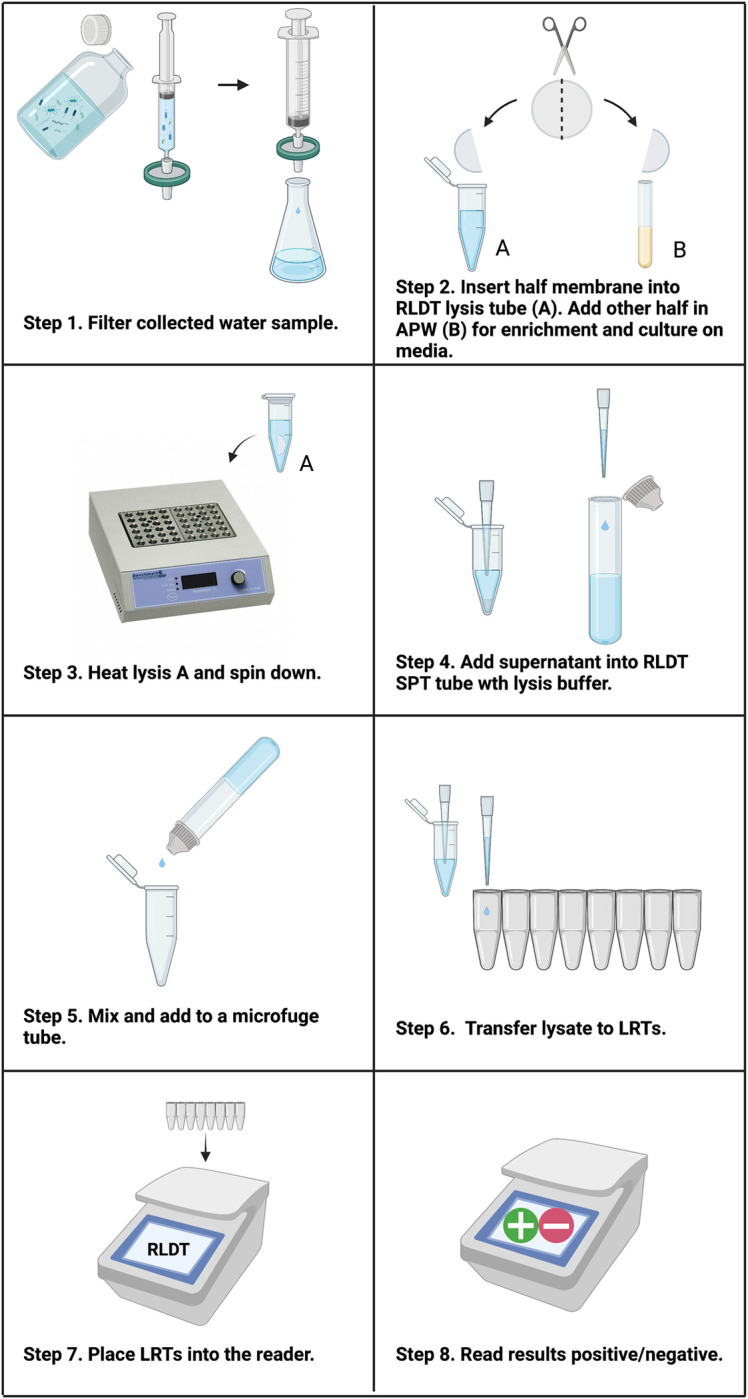
Workflow of cholera RLDT from water samples. Step 1, Filter 400ml water using a syringe filter. Step 2, cut membrane in half and insert one half in the lysis tube (A). Place the other half in APW media (B) for overnight enrichment at room temperature followed by culture on TCBS. Step 3, Heat tube A for 5 minutes in a heat block or water bath and spin down. Step 4, Transfer the supernatant from tube A into the SPT. Step 5, Squeeze the SPT tube to add the lysate to the lysis tube. Step 6, Add lysate from the lysis tube to LRTs. Step 7, Place the LRTs in the reader. Scan the barcode for the cholera RLDT program. Step 8, Read results displayed on the screen real time or after 40 minutes. The illustration was created with BioRender.com.

Four hundred mL of drinking or environmental water samples were filtered through 0.22μm PVDF membranes (Millipore Sigma Durapore, MA, USA) using a syringe (Becton, Dickinson and Company, NJ, USA). The membranes were cut in half using sterilized scissors, and one half was used for RLDT directly. The remaining half was placed in APW for enrichment for overnight at ambient temperature without shaking, followed by culture on TCBS agar. For RLDT, the sample on the PVDF membrane was heat lysed in the lysis tube with extraction lysis buffer and spin down, followed by processing of the supernatant through the SPT tube as described in Fig 2.

### Cholera RLDT kit

The cholera RLDT is prepared as a kit that includes the LRT strips and components required for sample preparation. Each LRT strip has six microcentrifuge tubes, which contain a lyophilized master mix. The first three microcentrifuge tubes were sequentially filled with the complete master mix targeting the O1*rfb*, *ctxA*, and reaction inhibitor control, while the remaining three microcentrifuge tubes were filled in the same sequential order so that each strip was used to test up to two samples. This arrangement of tubes could be changed to meet the user’s needs. For example, only *ctxA* gene and control could be used per sample, and therefore four samples could be tested at once using eight tubes strips.

The fluorometer reader in this study can incubate and read one LRT strip, up to eight targets simultaneously. This device is optimized for isothermal chemistry and allows real-time monitoring of amplification with a touch screen interface, data storage, a rechargeable battery, and handheld portability. The algorithm can be set depending on the need of end users for binary +/- results or in-depth analysis of the real-time amplification. The RLDT assay programs are coded in bar codes and scanned to include in the reader.

### Performance testing of cholera RLDT kit

The *V*. *cholerae* O1 El Tor strain N16961 was used in performing analytical sensitivity and specificity analysis of RLDT. The strain was cultured in LB broth and incubated at 37°C while shaking for 6 hours. The colony-forming units (CFU) were determined by optical density measurements and quantitative plate counts performed from the culture. Naïve stool samples obtained from the donors negative for cholera were aliquoted and spiked with 10-fold serially diluted cultures of cholera before being processed for RLDT. The spiked serially diluted stool samples were also tested after spotting and dried on filter paper.

For performance testing of cholera RLDT from water, samples were collected from the estuarine Inner Harbor in Baltimore, Maryland. Water samples were spiked with 10-fold serially diluted cultures of N16961 *V*. *cholerae* strain and processed as described before for RLDT.

### Statistical analysis

The coefficient of variations (CV) was calculated as the ratio of the standard deviation to the mean. All graphs and statistical analyses were performed using GraphPad, CA (Version 9) and Stata Corp LLC (Version 16) software.

## Results

### Performance specifications of cholera RLDT kit

#### Detection of cholera from stool samples using cholera RLDT kit

**Fig 3:**
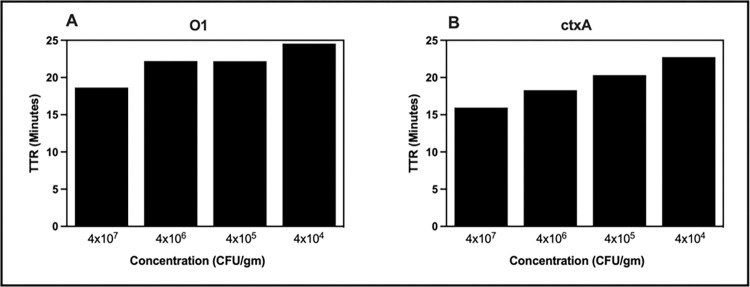
Lowest detection limit (LOD) of cholera RLDT tested directly from the stool. Stool samples were spiked with ten-fold serial dilutions of *V*. *cholerae* N16961. The spiked stool samples were processed and tested with the RLDT kit. LOD was determined as the lowest concentration at which the target gene was detected in all ten runs. A: O1*rfb* gene; B: *ctxA* gene.

The analytical sensitivity of cholera RLDT was determined by testing 10-fold serial dilutions of stool samples spiked with *V*. *cholerae* strain ranging in concentration from 40 to 4 x 10^7^ CFU/gm of stool using the RLDT kit. Each dilution was tested ten times. The lowest limit of detection (LOD) was defined as the lowest concentration at which the target gene could be detected in all ten runs. The LOD for the O1*rfb* gene and the *ctxA* gene was 4 x 10^4^ CFU/gm of stool (Fig 3). RLDT could detect cholera at a concentration lower than the LOD. At 4000 CFU/gm and 400 CFU/gm, RLDT could detect cholera 6 out 10 and 1 out of 10 times, respectively.

Analytical specificity was determined by testing reference strains with the cholera RLDT kit (Table 2). No amplification was observed for naïve stool or for pathogens other than the cholera O1 and *ctxA* strains indicating that the cholera RLDT assay is specific. Notably, the ETEC strains with LT were negative by cholera RLDT, showing the RLDT primers are specific for *ctxA*.

**Table 2:** List of strains used for the evaluation of the specificity of cholera RLDT.

Pathogen/Strain	Source	O1	*ctxA*
*Vibrio cholerae* O1 N16961 (El Tor Inaba)	ATCC	+	+
*Vibrio cholerae* O395 (O1 Classical)	India	+	+
*Vibrio cholerae* O1 IDH12695 (Ogawa)	India	+	+
*Vibrio cholerae* IDH00077 (non-O1 and non-O139)	India	-	-
*Vibrio cholerae* IDH04713 (non-O1 and non-O139)	India	-	-
*Vibrio cholerae* NT4669 (non-O1 and non-O139)	India	-	-
*Vibrio cholerae* IDH01793 (non-O1 and non-O139)	India	-	-
*Vibrio cholerae* O139 IDH00502	India	-	+
*Vibrio cholerae* O139 SG24	India	-	+
*Vibrio cholerae* O139 OR18	India	-	+
ETEC H10407 (LT, ST)	WRAIR	-	-
ETEC B7A (LT, ST)	WRAIR	-	-
ETEC E24377A (LT, ST)	WRAIR	-	-
*E*. *coli* 25922	ATCC	-	-
*E*. *coli* 042 (EAEC)	ATCC	-	-
*E*. *coli* 2348/69 (EPEC)	ATCC	-	-
CR100/C5/RS(C1)	Bangladesh	-	-
*Shigella flexneri* 2a 2457T	WRAIR	-	-
*Shigella boydii* AMC 4006	ATCC	-	-
*Shigella sonnei 53G*	WRAIR	-	-
*Campylobacter jejuni* 33291	ATCC	-	-
*Staphylococcus aureus*	ATCC	-	-
*Salmonella enterica* serovar Typhimurium 700720DQ	ATCC	-	-

Repeatability was tested by performing ten repeats of each of the two stool samples spiked with cholera strain, one with 10^7^ CFU and the other with 10^5^ CFU of cholera strain/gm of stool. Reproducibility was tested using ten identically spiked stool samples with either 10^7^ CFU or 10^5^ CFU of cholera strain/gm of stool over three days. We observed positive results for all (100%) of the repeatability and reproducibility assays that were tested. During the repeatability and reproducibility experiments, we determined the % CV of the TTR values of the *ctxA* and O1*rfb* genes which ranged from 4.6% to 6.3% for *ctxA* and 8% to 13.4% for O1*rfb* (Table 3). To understand if cholera RLDT could be used as a semi-quantitative assay, we analyzed the linearity of the TTR values of *ctxA* gene using serially diluted spiked stool samples. Linearity was established by averaging the TTR over 10 runs of each dilution and plotting the average results. The TTR increased consistently as the concentration of the bacteria in the samples decreased. The R^2^ linearity value was 0.999 and the linear regression equation y = 2.237x+13.745.

**Table 3:** Variations in cholera RLDT Time to Result (TTR) in repeatability and reproducibility analysis.

Targets	TTR CV (%)			
	Repeatability		Reproducibility	
	10^7^CFU/gm of stool	10^5^CFU/gm of stool	10^7^CFU/gm of stool	10^5^CFU/gm of stool
O1*rfb*	13.4	11.3	8.0	12.3
*ctxA*	5.9	4.6	5.1	6.3

**Fig 4:**
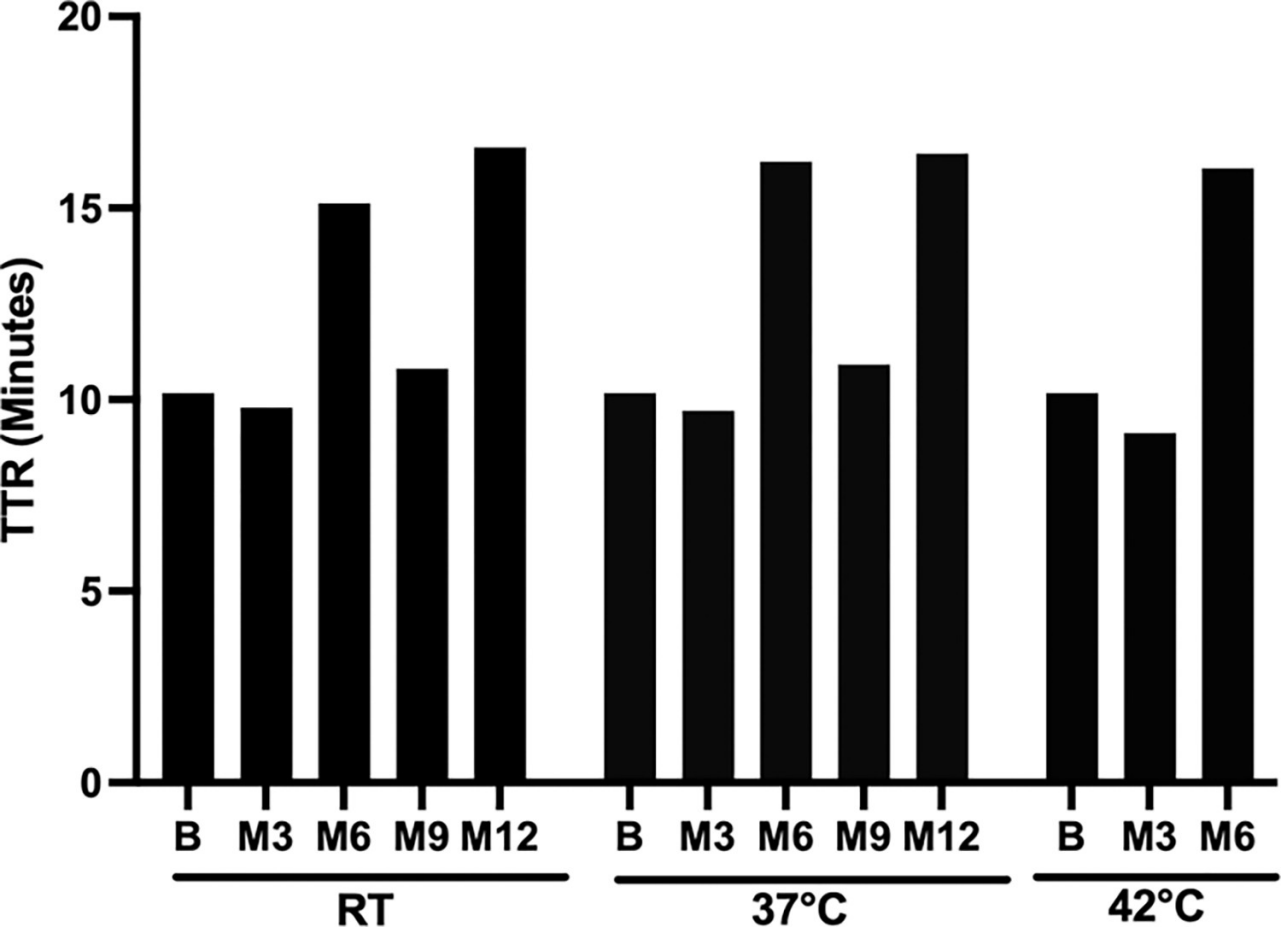
Stability of the dry cholera RLDT. The cholera lyophilized reaction tubes (LRT) strips were stored at room temperature (RT), at 37°C, and 42°C. LRT strips were tested using the RLDT kit at three months intervals and the Time to Results (TTR) were recorded.

To evaluate the stability of the dry RLDT formulations, the dry LRT strips were incubated at room temperature (~23°C) and 37°C for one year and 42°C for six months and tested at the day 0 and every three months using the RLDT kit. Dry RLDT formulations were stable and functional at ambient temperature and 37°C for one year and 42°C for six months (Fig 4). All the TTRs were within 17 minutes.

### Detection of cholera from dried stool spots on filter paper using cholera RLDT kit

**Fig 5:**
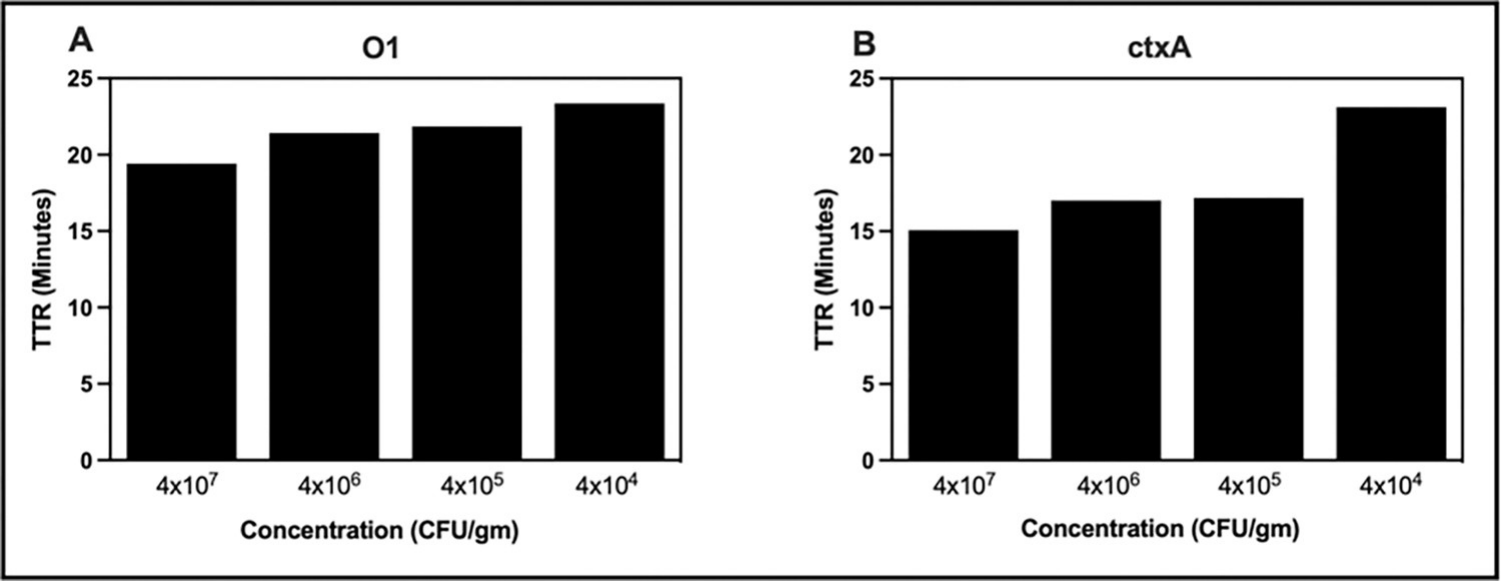
Lowest Detection Limit (LOD) of cholera RLDT from dried stool on filter paper. Stool samples were spiked with ten-fold serial dilutions of *V*. *cholerae* N16961and 50 uL of each dilution was spotted on filter paper. The stool spots were processed and tested with an RLDT kit. LOD was determined. A: O1*rfb* gene; B: *ctxA* gene.

We tested cholera RLDT using serial dilutions stool spiked with *V*. *cholerae* ranging from 40 to 4 x 10^7^ CFU/gm of stool spotted and dried on filter paper and determined the LOD. The samples were prepared and tested as previously described. The LOD for the O1*rfb* gene and the *ctxA* gene was 4 x 10^4^ CFU/gm (Fig 5). At 4 x 10^3^ CFU/gm, RLDT could detect cholera 4 out of 10 times, while cholera could not be detected at a concentration of 400 CFU/gm.

### Detection of cholera from environmental water samples using cholera RLDT kit

**Fig 6:**
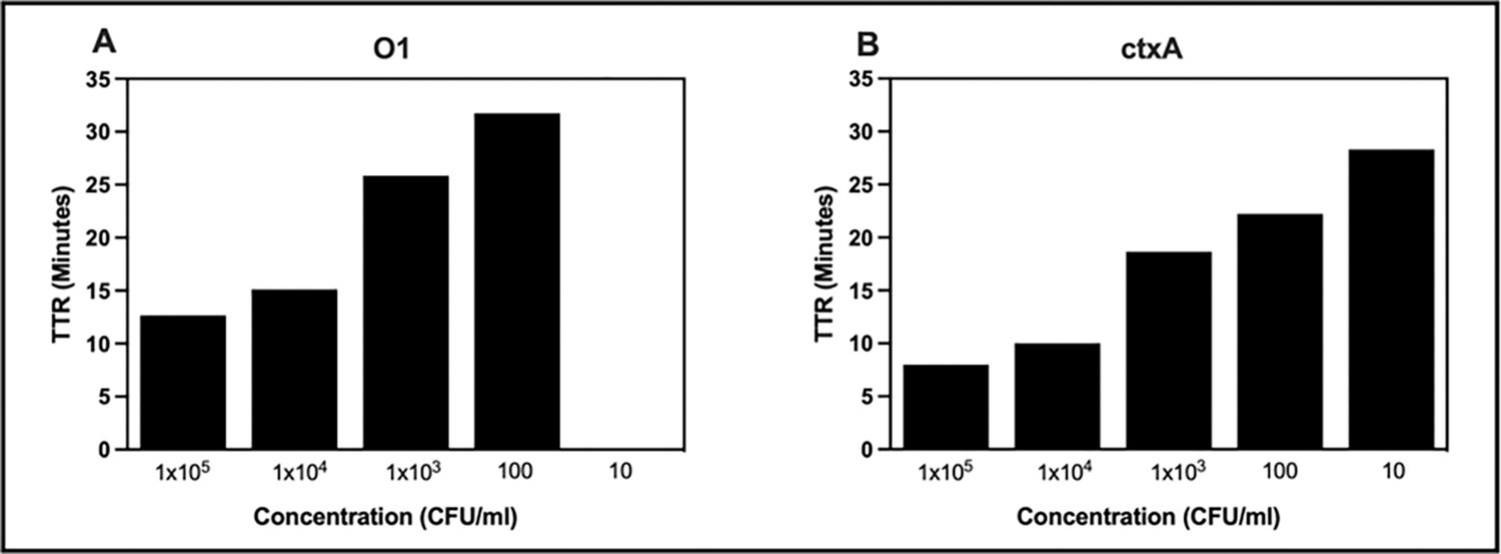
Lowest detection limit (LOD) of cholera RLDT for environmental water samples. Environmental water samples were spiked with ten-fold serial dilutions of *V*. *cholerae* N16961 and processed and tested with an RLDT kit. LOD was determined. A: O1*rfb* gene; B: *ctxA* gene.

We tested cholera RLDT to detect *V*. *cholerae* in environmental water samples and determined the LOD. Environmental water samples spiked with serial dilutions of *V*. *cholerae* strains with the concentrations ranging from 1x10^5^ to ~1 bacterium per ml were prepared and tested with RLDT kit directly, without enrichment as previously described. The LOD was 100 CFU/ml of environmental water without enrichment for the O1*rfb* gene and 10 CFU/ml for the *ctxA* gene (Fig 6).

### Advantages of the cholera RLDT

RLDT is performed directly from the stool samples with minimum treatment. Using the rapid sample preparation and dry formulation, the assay is simple. There is a minimum hands-on time of <5 minutes for processing the sample and adding the lysates to the LRTs. The assay results are read as +/- using a battery-operated handheld reader. The RLDT kit can be stored at ambient temperature and thus can avoid maintaining a cold chain. The RLDT kit provides all the reagents and supplies required and could mitigate the constraints in obtaining reagents, primers, and plastics at the LMICs. The LRTs are already filled with dry reagents and primers, avoids individual addition of each reagent, minimizing contaminations and simplify the process for the users. The assay is rapid, taking <50 minutes from stool to result. As six primers are used for detecting each target, RLDT has high specificity. RLDT only requires a heat block and a reader, which is optional. The assay generates minimum biohazard wastes. The cholera RLDT detects both the O1 and cholera toxin gene and therefore if a new cholera toxigenic strain emerges, will be able to detect immediately. This assay can be performed from stool, dried stool on filter paper, environmental and drinking water.

## Discussion

The novel RLDT assay for detecting cholera is a rapid, simple, and sensitive nucleic acid amplification-based diagnostic assay. RLDT assay could provide a point-of-care diagnostic for cholera in areas with limited access to adequate laboratory infrastructure. This assay was designed for maximum ease of use with transport and storage at ambient temperature. The stool is a difficult substrate because of the presence of inhibitors that could inhibit amplification. We developed a novel technique for a simple, rapid, and minimum hands-on time stool sample processing procedure which maintains high sensitivity of the assay, LOD of 10^4^ CFU/gm of stool. This LOD is much lower (more sensitive) than the current lateral flow RDTs [9] and similar to the cholera *tcpA* gene in the TaqMan Array Card for enteropathogen detections [28] that has been used in the reanalysis of the samples from the Global Enteric Multicenter Study (GEMS) [29] and the multisite birth cohort study (MAL-ED) [30]. Of note, the TaqMan Array card uses purified DNA, and RLDT is performed directly from the stool.

RLDT could be performed also from stool samples dried on filter papers, which allows storing samples for later testing and, if required, sending samples from remote areas or areas with political conflicts to a better facility.

Environmental reservoirs and contaminated drinking water with *V*. *cholerae* leave millions at risk of cholera. During cholera outbreaks, promptly identifying the contaminated water source could prevent numerous additional infections and thus could save lives. With RLDT, cholera could be detected from the environmental or drinking water with high sensitivity within an hour.

Unlike the current lateral flow assays [10,11] which only detects O1and O139 antigens, the cholera RLDT detects both the O1 and cholera toxin genes and therefore if a new toxigenic cholera strain emerges (as O139 cholera strain emerged and caused pandemic), will be able to detect immediately.

The cholera RLDT assay is made in a dry format where all the reagents are lyophilized, which avoids maintaining a cold chain during shipment and storage. This dry format also avoids handling individual reagents by the end users, who only need to add the processed sample to the LRT strips. RLDT kit includes all the reagents and plastics required for the assay, which minimizes the difficulty of procuring reagents and plastics by the end users, which is often a challenge in endemic countries.

As LAMP, the RLDT results can be read by the naked eye or using a UV illuminator. To avoid end-user bias when using the assay by the hospital staff or in the field or at the community level by the technicians with minimum or no laboratory training, we preferred to use the handheld battery-powered fluorometer reader, which can read the results as positive or negative. Although this equipment adds additional cost to the assay, it is only a one-time primary investment and could be used for RLDT assays for other pathogens, that we developed [27,31,32].

Although, RLDT is a qualitative test, the TTR of RLDT has a linear relation with the CFU of the bacteria per gram of stool. Thus, RLDT can semi-quantify the number of the target bacteria in the stool.

Since RLDT assay is rapid, stool samples that are positive by RLDT for cholera, could be sent to the laboratory for culture on the same day to isolate colonies for downstream characterization of the strains, e.g., whole genome sequencing.

Several LAMP-based assays were developed previously in the laboratories for the diagnosis of cholera [9,33–43]. The stool is a complex sample to extract DNA and amplify because of the presence of inhibitors. The LAMP assays previously developed to detect cholera from stool are either from colonies isolated from culturing the stool or from purified DNA extracted from stool with commercial kits or a complex process that is not feasible at low resource settings. In addition, these assays require maintaining a cold chain, which is difficult to achieve in these settings. RLDT has addressed these issues and adapted LAMP to be more applicable to the endemic settings where it is most needed. One could backpack the RLDT kit and the reader to perform the assay in a cholera outbreak setting to facilitate rapid results with high sensitivity and specificity.

In conclusion, cholera RLDT has several advantages, including rapid results, simple operating procedures, easy readout of the results, and high analytical sensitivity, equivalent to quantitative PCR. In addition, RLDT is electricity and cold-chain-free. All these qualities make RLDT easy to scale up and appropriate to use in endemic settings. RLDT assay for detection of ETEC was feasible to implement in an endemic country setting, Zambia [32].

Field evaluations of cholera RLDT are currently underway in the South Asian and African countries to determine the feasibility of applying this assay in the endemic settings and compare the performance of the RLDT to the reference diagnostic assays of cholera.
